# A Review of Recombinase-Aided Amplification (RAA) in Veterinary Medicine

**DOI:** 10.3390/microorganisms14102352

**Published:** 2026-10-14

**Authors:** Shaomei Li, Jieqi Gao, Yongqiang Miao, Qianlin Chen, Hao Mu, Hua Zheng, Yifan Zhang, Jie Zhou, Chunhua Huang, Mingwen Li, Yuandi Yu, Liu Yang

**Affiliations:** 1 Chongqing Academy of Animal Sciences, Chongqing 402460, China; 2 National Technology Innovation Center for Pigs, Chongqing 402460, China; 3 Rongchang, Animal Disease, Observation and Research Station, Ministry of Agriculture and Rural Affairs, Chongqing 402460, China; 4 Chongqing Yubei District Animal Husbandry and Fisheries Station, Chongqing 402460, China; 5 Shizhu Tujia Autonomous County Animal Husbandry Industry Development Center, Chongqing 409100, China

**Keywords:** recombinase-aided amplification (RAA), veterinary diagnostics, pathogen detection, point-of-care testing (POCT), isothermal amplification

## Abstract

Rapid and accurate pathogen detection is crucial to mitigating the significant economic impacts of infectious animal diseases. Recombinase-aided amplification (RAA) has emerged as a highly promising isothermal diagnostic tool, characterized by its rapid turnaround time, robust amplification efficiency, and single-copy analytical sensitivity. In veterinary diagnostics, RAA has evolved from single-pathogen proof-of-concept assays to become a promising technology for point-of-care scenarios. This review critically evaluates the integration of RAA with real-time fluorescence, CRISPR-Cas systems, lateral flow dipsticks (LFDs), and suspension microarray technologies, highlighting their impact on assay sensitivity and point-of-care (POC) applicability. Despite recent advancements, including encouraging progress in multiplexing and expanded applications in companion animal medicine, inherent challenges such as matrix effects in crude clinical samples, aerosol contamination risks, and commercialization barriers remain. By benchmarking RAA against traditional molecular methods and structurally similar technologies like recombinase polymerase amplification (RPA), this review outlines future trajectories for optimizing RAA platforms, aiming to maximize their utility in routine veterinary surveillance and epidemiological control.

## 1. Introduction

Rapid and accurate pathogen detection is pivotal for effective epidemic containment in veterinary medicine. Cumbersome protocols and misdiagnosis of co-infections often exacerbate economic and clinical losses. Traditional detection methods including microscopic examination, microbial culture and serological testing provide complementary diagnostic information alongside molecular diagnostics, although they are often time-consuming, technically demanding and limited for early-stage infection detection [1]. Consequently, establishing a diagnostic system that is rapid, efficient, user-friendly, yet sensitive has become a major focus for researchers in the veterinary diagnostics field.

**Figure 1:**
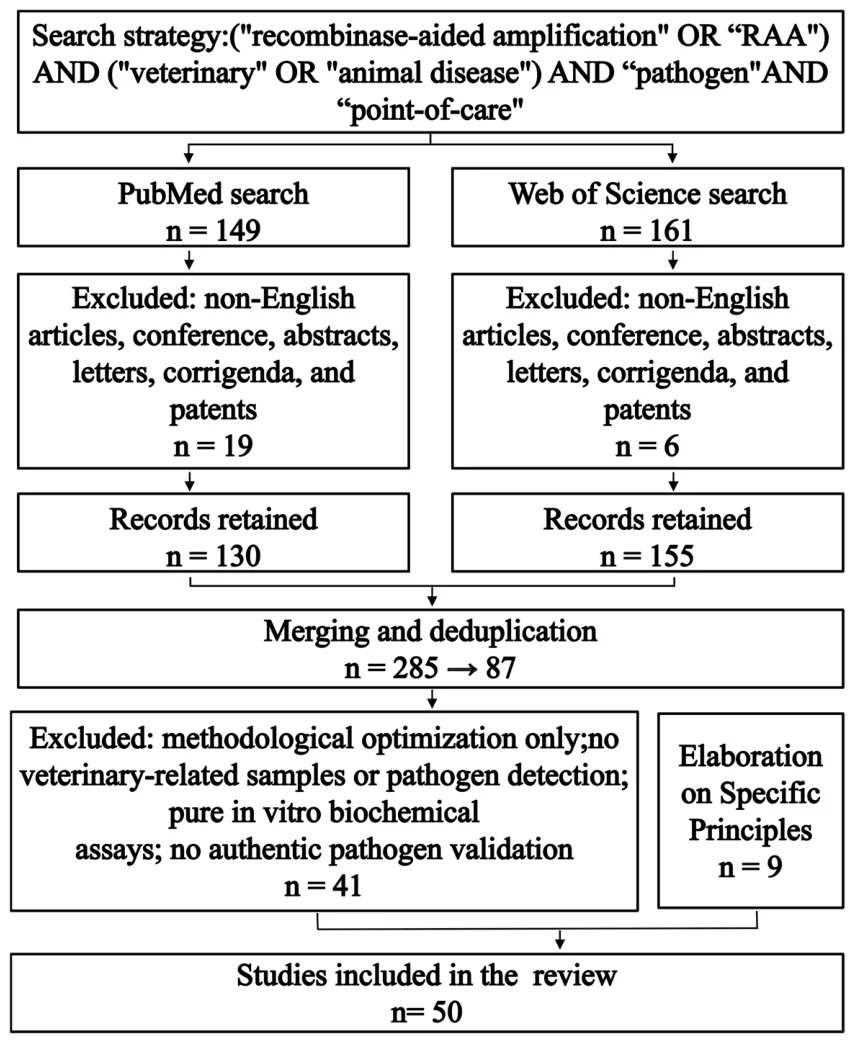
Flow diagram of the literature screening. After searching PubMed and Web of Science and excluding studies that did not meet the requirements, nine principle-focused articles were additionally included, and 50 studies were ultimately included.

In recent years, isothermal amplification technologies such as loop-mediated isothermal amplification (LAMP), recombinase polymerase amplification (RPA), and recombinase-aided amplification (RAA) have gradually evolved into important technical tools for rapid pathogen screening [2,3]. These technologies do not require a thermocycler and have low instrument–equipment requirements [4,5]. Among these, RAA enables amplification under near-physiological temperatures of 37–42 °C within only several minutes, and exhibits favorable application potential among isothermal detection platforms [3]. To evaluate the application of recombinase-aided amplification (RAA) in veterinary pathogen detection, we conducted a systematic literature search in PubMed and Web of Science for studies published through March 2026. The search strategy combined the following key terms: (“recombinase-aided amplification” OR “RAA”) and (“veterinary” OR “animal disease”) and “pathogen” and “point-of-care”. Non-English articles, conference abstracts, letters, corrigenda, and patents were excluded. After merging and deduplication, studies limited to methodological optimization and assays lacking authentic pathogen validation were further excluded. Ultimately, 50 studies meeting the inclusion criteria were included, encompassing pathogens associated with livestock, poultry, companion animals, aquatic species, parasitic infections, and zoonotic vectors (Figure 1).

This review systematically elaborates on the molecular mechanisms and performance of RAA, summarizes its application progress in veterinary diagnostics, and dissects the integration strategies of RAA with CRISPR, microfluidics, and immunochromatography. Furthermore, it systematically compares RAA with other detection methods, aiming to provide a reference for the development of robust diagnostic solutions for animal diseases.

## 2. Development and Application of RAA Technology

### 2.1. Principles of Basic RAA

**Figure 2:**
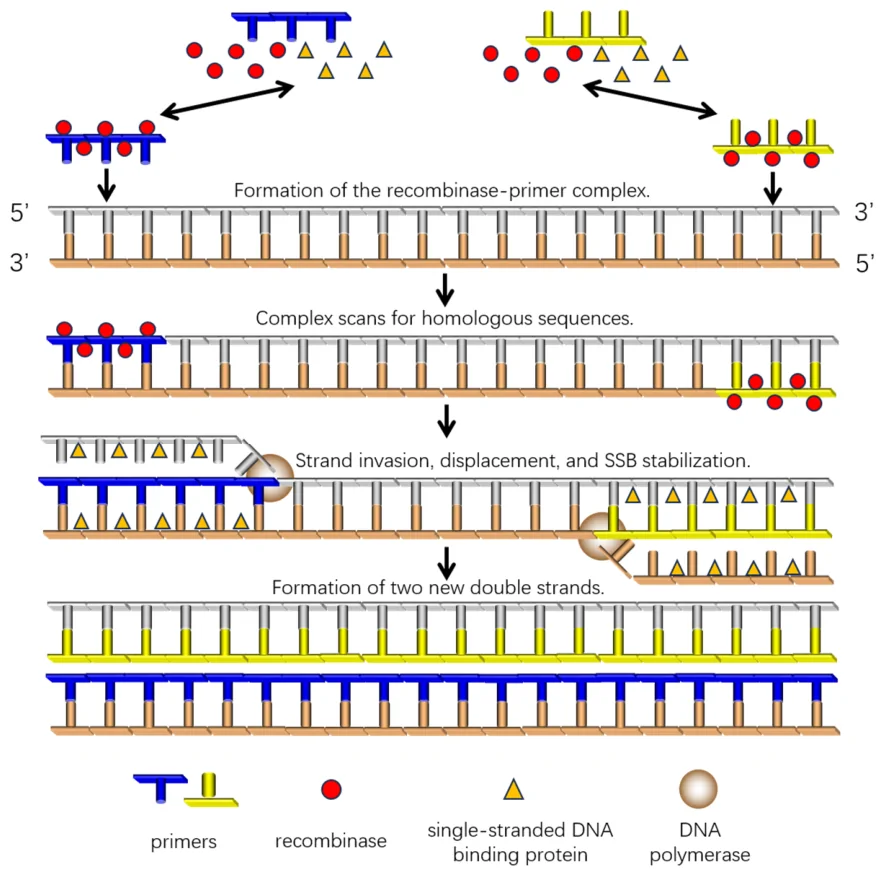
Schematic representation of the RAA reaction mechanism. The recombinase forms a nucleoprotein complex with primers to scan double-stranded DNA for homologous target sequences. Upon strand invasion, SSB stabilizes the displaced single strand. Subsequent dissociation of the recombinase allows DNA polymerase to initiate complementary strand synthesis from the 3′ end of the primers.

RAA is an isothermal nucleic acid amplification technique that utilizes a recombinase. Upon initiation of RAA, the recombinase binds to primers to form a recombinase–primer nucleoprotein filament. The filament performs a homology search along the double-stranded DNA template and, upon recognition of the complementary target sequence, mediates strand invasion, generating a D-loop structure. Following strand invasion, SSB binds to and stabilizes the displaced strand, preventing re-annealing. The 3′-OH end of the primer is then accessible for DNA polymerase, which extends the nascent strand while displacing the recombinase. A DNA polymerase with strand-displacement activity subsequently initiates synthesis from this 3′-OH end to extend the nascent strand [5,6] (Figure 2). The entire amplification process can yield detectable levels of target DNA within 5–20 min. Analysis of the basic RAA products by agarose gel electrophoresis reveals specific amplification bands for Salmonella even when the template DNA concentration is as low as 10^5^ copies/μL [7]. However, the reliance on end-point, open-tube readouts significantly increases the risk of aerosol contamination, driving the evolution of RAA modifications.

### 2.2. Reverse Transcription RAA (RT-RAA)

**Figure 3:**
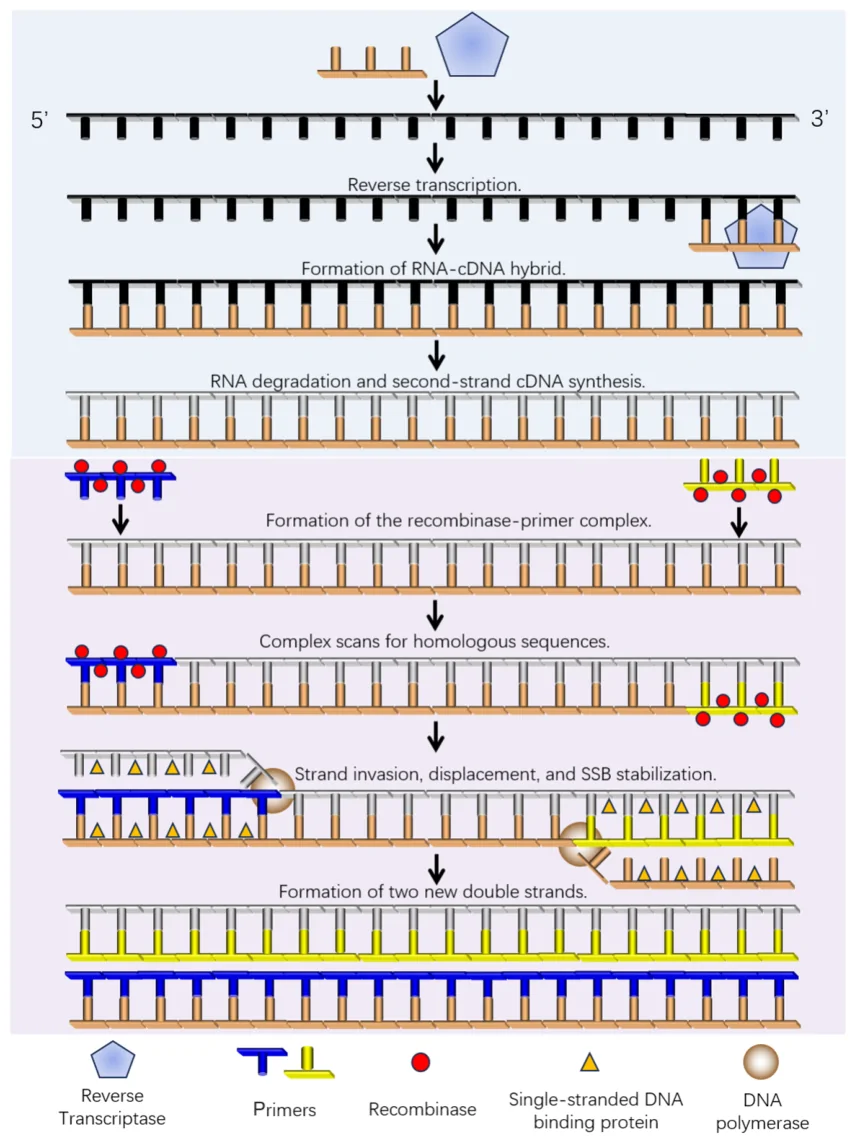
Schematic diagram of the RT-RAA reaction workflow. In a one-pot system, reverse transcriptase first converts the viral RNA template into cDNA, involving reverse transcription, formation of an RNA-cDNA hybrid, RNA degradation and second-strand cDNA synthesis. Subsequently, the RAA proceeds: recombinase binds with primers to form nucleoprotein complexes, which scan the target sequence, mediate strand invasion and strand displacement; single-stranded DNA binding protein (SSB) stabilizes the displaced strand, and DNA polymerase completes the synthesis of new complementary double strands.

Compared with standard RAA, RT-RAA additionally introduces a reverse transcriptase (RT) into the core RAA enzyme system. During the initial stage of the reaction, the reverse transcriptase uses the viral RNA genome as a template and the RAA-specific primer as the reverse transcription primer to synthesize cDNA, which then serves as the template for the subsequent RAA [8] (Figure 3). RT-RAA assays have achieved exceptional analytical sensitivity for highly contagious enteric viruses, detecting porcine epidemic diarrhea virus (PEDV) at 1 copy/μL [9] and porcine group A rotavirus (PoRVA) at 7 copies/μL within 10 min [10]. Furthermore, it exhibits broad diagnostic applicability, successfully identifying avian metapneumovirus subtype C (aMPV-C) [11] and Newcastle disease virus (NDV) [12]. Despite its rapidity and potential suitability, basic RT-RAA lacks real-time quantitative capabilities, necessitating further technological integration.

### 2.3. Real-Time Fluorescence-Based RAA (RF-RAA)

**Figure 4:**
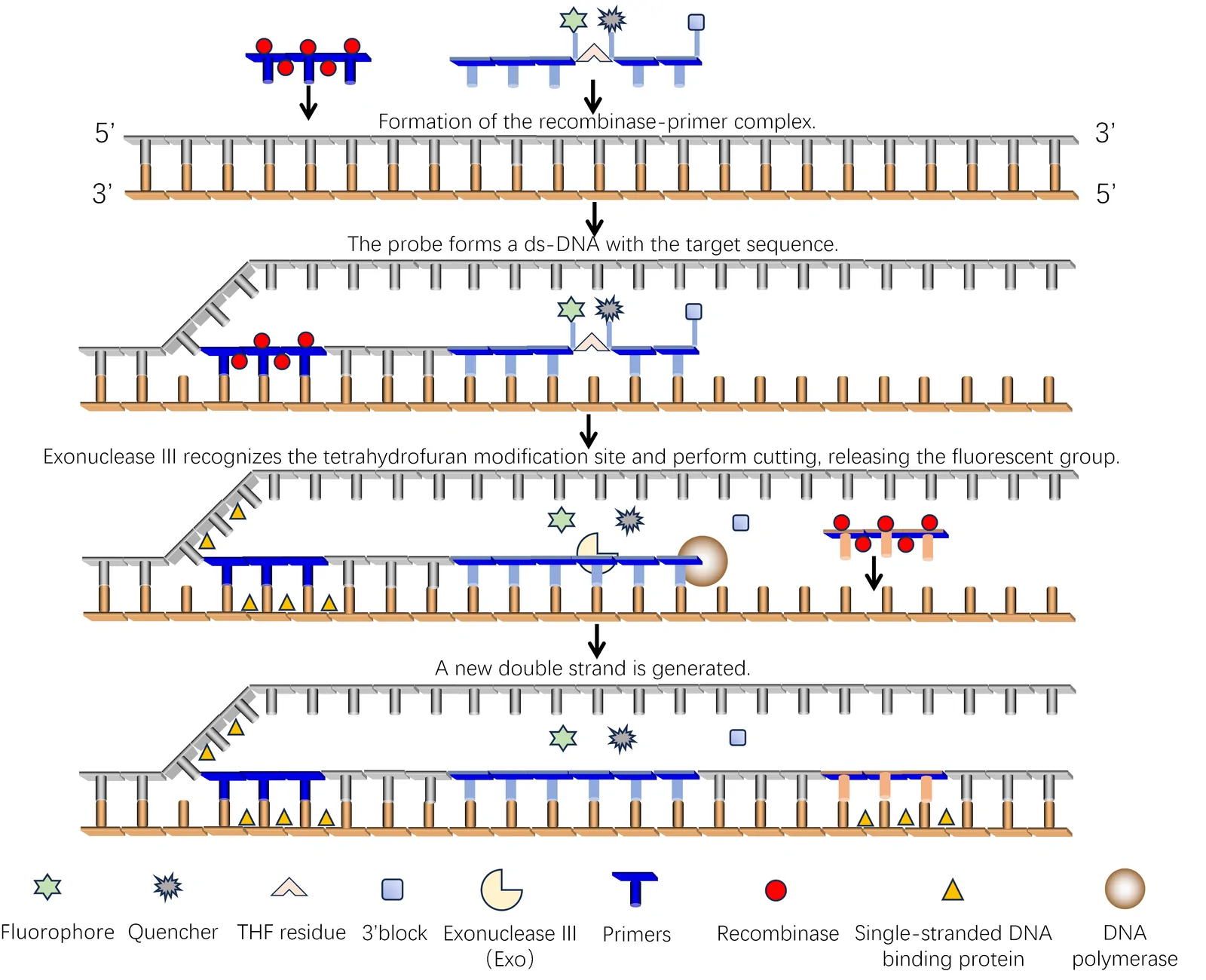
Schematic mechanism of RF-RAA. The Exo probe contains a 5′ fluorophore, an internal tetrahydrofuran (THF) basic site, and a 3′ quencher with a 3′ blocking group. Upon specific hybridization with the amplified target DNA to form a duplex, Exo III recognizes and cleaves the THF site, separating the fluorophore from the quencher and generating a real-time fluorescent signal that increases as amplicons accumulate; primer extension by the strand-displacing DNA polymerase then generates a new double-stranded DNA product.

To achieve real-time monitoring of amplification and reduce the risk of aerosol contamination, researchers have developed the real-time fluorescence recombinase-aided amplification (RF-RAA) assay, adapted from the probe-based real-time fluorescence readout originally developed for recombinase-based amplification [13]. Three major probe-chemistry formats are available for RF-RAA: Exo probes, Nfo probes, and fpg probes. The Fpg probe system, which employs formamidopyrimidine DNA glycosylase (Fpg), represents an alternative chemistry but is less commonly used in RF-RAA. The Nfo probe system relies on endonuclease IV (Nfo), which is mostly coupled with lateral-flow dipstick endpoint detection and enables visual readout [14,15]. In contrast, the Exo probe system utilizes exonuclease III (Exo III), which also cleaves the AP site generated by THF modification, and is widely adopted for real-time fluorescent signal acquisition (Figure 4). In the Exo probe, a fluorophore and a quencher are positioned flanking the THF site; only when the probe specifically hybridizes to its complementary amplified strand to form a duplex, with the bases flanking the THF site paired, does Exo III cleave the AP site, physically separating the fluorophore from the quencher and generating a fluorescent signal [16].

In veterinary practice, RF-RAA accurately differentiates closely related subtypes, such as avian influenza virus (AIV) H5, H7, and H9 strains [17], and enables simultaneous multiplex detection of emerging swine pathogens, such as porcine circoviruses (PCV3/PCV4) [18]. Furthermore, it detects bacterial pathogens like Streptococcus equi at a threshold of 10 copies/μL within 20 min [19]. Recently, the integration of novel nucleases, including a double-stranded DNA-specific nuclease (dsDNase) [20] and the programmable Pyrococcus furiosus Argonaute (PfAgo) [21], has further suppressed non-specific background amplification, pushing the sensitivity limits for pathogens like porcine deltacoronavirus (PDCoV).

### 2.4. CRISPR-Cas12a/Cas13a Assisted RAA Method

**Figure 5:**
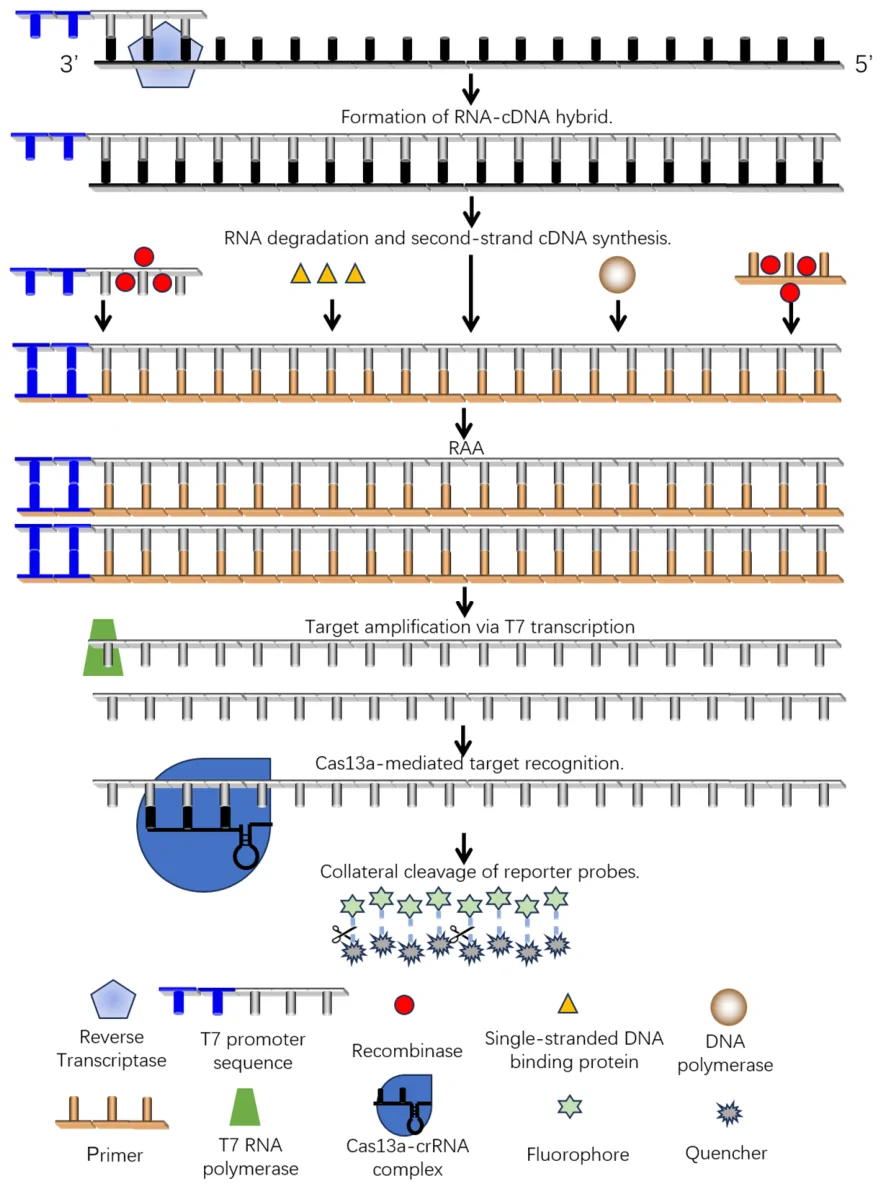
Schematic workflow of RAA-CRISPR/Cas13a dual-amplification detection. First, reverse transcription converts the initial RNA template into cDNA, followed by RAA. Modified primers incorporate the T7 promoter sequence into RAA amplicons. T7 RNA polymerase then drives in vitro transcription to produce large amounts of target single-stranded RNA. Afterwards, Cas13a-crRNA complexes recognize and bind the target RNA, which activates the collateral RNase activity of Cas13a. The activated Cas13a non-specifically cleaves surrounding reporter probes, separating fluorophores from quenchers and producing detectable fluorescence signals.

The combination of RAA with CRISPR-Cas12a/Cas13a systems forms a pre-amplification and target-activated signal-amplification workflow. RAA acts as a rapid pre-amplification module to enrich target nucleic acids, while the crRNA-guided CRISPR-Cas complex serves as a high-specificity secondary recognition step. CRISPR-Cas achieves signal amplification solely by cleaving reporter probes. Upon target recognition, the Cas enzyme exerts non-specific collateral-cleavage activity on surrounding reporter probes and generates strong readout signals [22,23] (Figure 5). Cas13a targets RNA substrates without PAM constraints, though it exhibits protospacer flanking site (PFS) preferences that can influence target selection. Nevertheless, the vast majority of RT-RAA-CRISPR/Cas13a workflows require the introduction of a T7 promoter and can be affected by the intrinsic sequence preference of the Cas13a enzyme [24,25].

RT-RAA-CRISPR/Cas12a coupled with lateral-flow dipsticks enables visual detection of Pigeon Rotavirus A (PiRVA) [26], Nipah virus (NiV) [27], and *Trichomonas gallinae* [28]. Comparable assays allow visual identification of peste des petits ruminants virus (PPRV) [29] and *Mycoplasma hyopneumoniae* (Mhp) [30]. For African swine fever virus (ASFV), no cross-reactivity was observed for this Cas12a-assisted system against the limited test panel of common porcine pathogens [31]. RT-RAA-CRISPR/Cas13a has successfully detected porcine reproductive and respiratory syndrome virus (PRRSV) [32] and bovine coronavirus (BCoV) [33]. Single-base discrimination can be accomplished with this technology under optimised experimental conditions. This capability is heavily dependent on crRNA design, mismatch-site position, and reaction parameters, and it is critical for differentiating closely related viral subtypes and identifying specific point mutations [34].

### 2.5. Lateral-Flow Dipstick (LFD) Integration with RAA Technology

**Figure 6:**
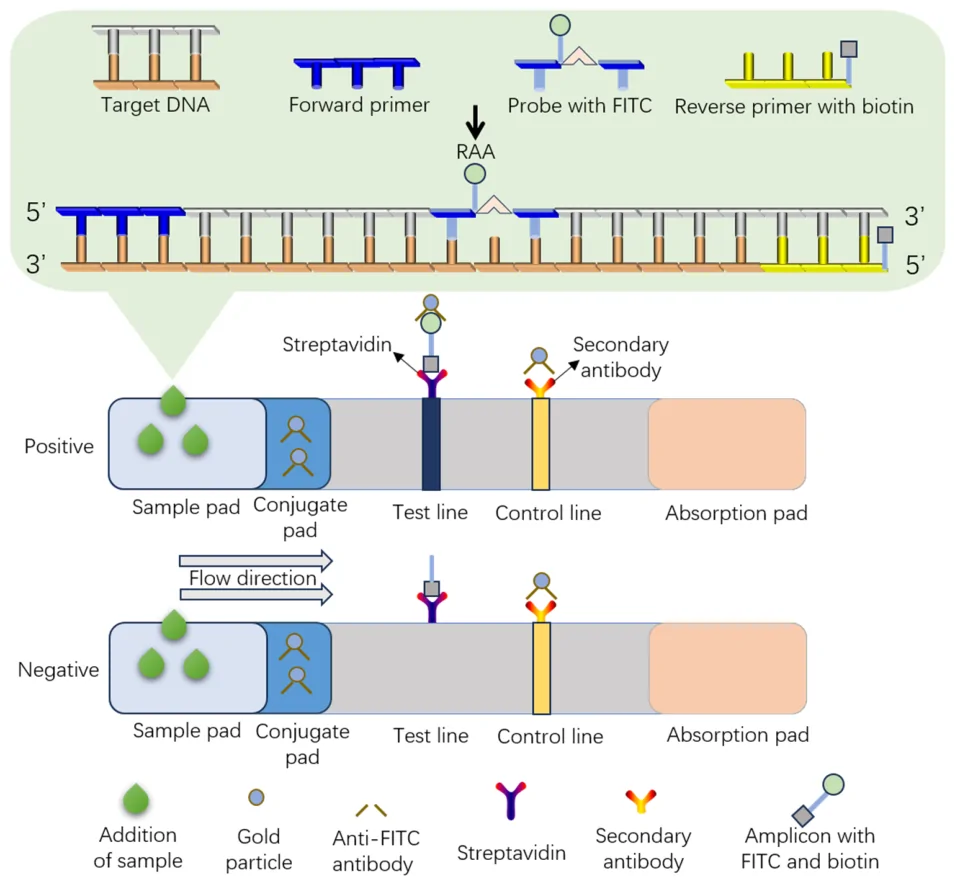
The mechanism of RAA-LFD. Demonstrates the instrument-free POCT solution. By dual-labeling amplicons with FITC and biotin, target sequences bound to gold-conjugated antibodies are captured by streptavidin at the test line, while excess gold conjugates are captured by secondary antibodies at the control line on a nitrocellulose membrane for visual readout.

LFDs provide an ideal instrument-free readout for POCT. By incorporating FITC- and biotin-labeled primers or probes into RAA amplicons, target sequences can be visualized via capillary flow and antigen–antibody recognition on a nitrocellulose membrane within minutes [15] (Figure 6). Multiplexing in isothermal amplification is often challenged by primer-dimer competition and ATP depletion. However, encouraging progress has been achieved with the development of a Triplex RAA-LFD assay, enabling the simultaneous detection of BCoV, IBRV, and BVDV from a single tube [35]. An RAA-LFD assay has been developed for efficient and accurate detection of porcine parvovirus [36]. Furthermore, the implementation of RAA-LFD has lowered the technical barrier for the rapid diagnosis of highly contagious diseases, such as Foot-and-Mouth Disease Virus (FMDV) [37]. Beyond simple detection, this platform has also been successfully extended to the rapid genotyping of Feline Coronavirus (FCoV) [38], demonstrating its versatility in clinical veterinary diagnostics. It should be noted that conventional open-tube RAA-LFD requires opening the reaction tube after amplification, which brings potential risks of amplicon aerosol contamination. Closed-cartridge sealed transfer systems are required to mitigate such hazards for true point-of-care applications.

### 2.6. Suspension Microarray and Other Unifying Technologies

**Figure 7:**
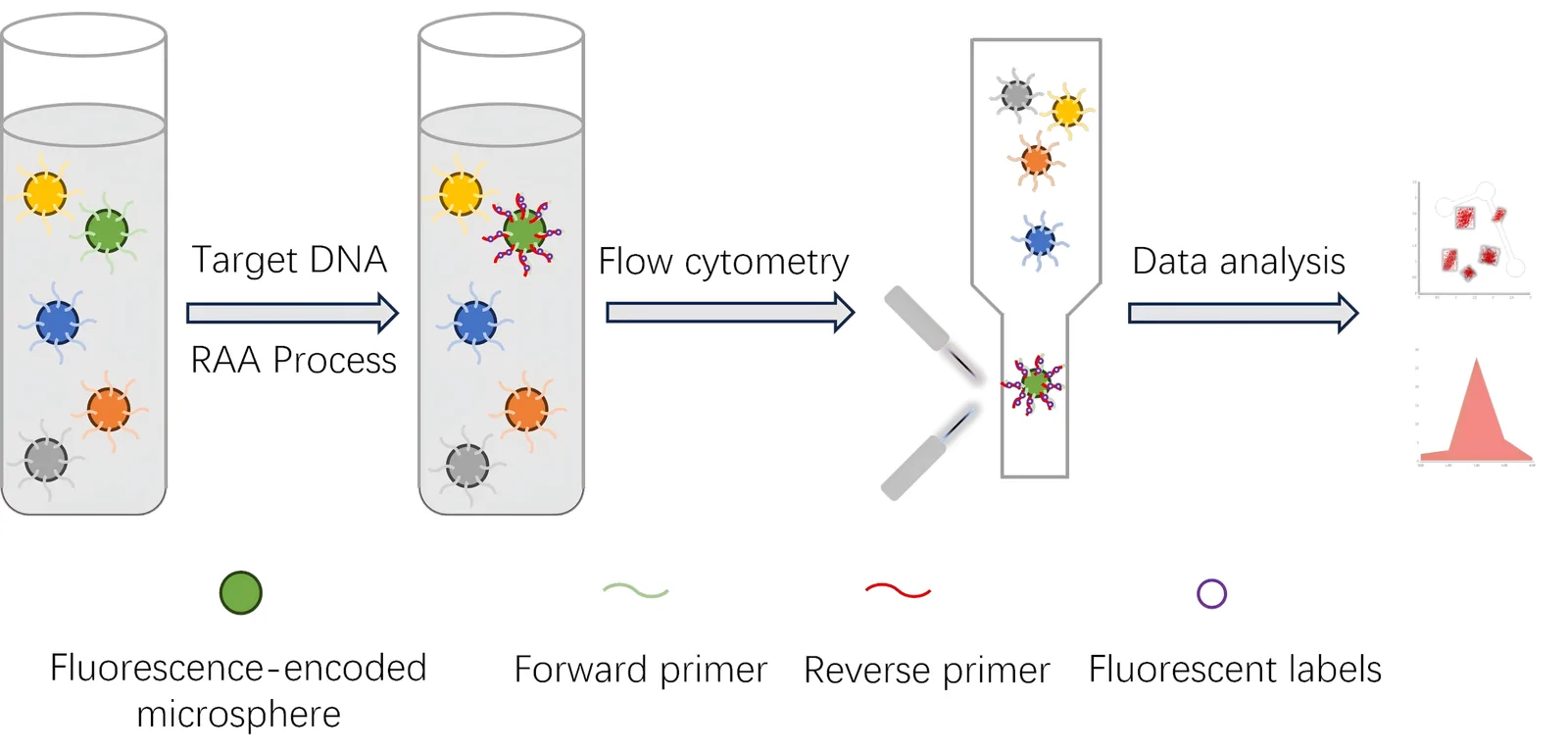
Schematic illustration of RAA coupled with suspension microarray technology. Fluorescence-encoded microspheres are conjugated with forward and reverse primers. During the RAA process, target DNA is amplified on the surface of the microspheres, generating fluorescently labeled amplicons. The microspheres are then classified and analyzed by flow cytometry, enabling highly multiplexed detection. Finally, data are processed and visualized as scatter plots and histograms.

To maximize clinical utility, RAA technology is evolving along high-throughput and ultra-portable trajectories. For large-scale epidemiological screening, RAA coupled with suspension microarray technology (employing fluorescence-encoded microspheres) takes advantage of flow cytometry-based bead classification to support highly multiplexed detection (Figure 7). Conversely, RAA has been adapted to interface with ubiquitous consumer electronics, such as using personal glucose meters (PGMs) for rabies virus (RABV) detection [39].

To systematically clarify the applicability of various RAA-derived techniques, this study summarizes and compares the diagnostic performance of different RAA-based assays for the rapid detection of veterinary pathogens, as shown in Table S1. This table covers multiple mainstream RAA detection formats, including conventional basic RAA, RF-RAA, CRISPR-RAA, LFD-RAA, and other novel integrated RAA platforms. Core analytical parameters are comprehensively evaluated, involving target genes, template types, reaction temperature and duration, and detection sensitivity. The compiled data encompass a broad spectrum of veterinary pathogens, including swine, avian, bovine, and aquatic viruses, as well as pathogenic bacteria and parasites, which fully demonstrates the favorable compatibility and extensive application potential of RAA technology in rapid pathogen detection.

Table S2 presents the clinical diagnostic performance of RAA assays targeting various veterinary pathogens, developed in different formats, including single-plex, multiplex, fluorescence-based and lateral flow readout. These assays were validated using diverse clinical specimens with conventional reference methods such as qPCR, RT-qPCR, and microbial isolation. Under clinical sample testing conditions, most RAA assays showed good agreement with standard detection methods.

## 3. Comparison of RAA with Other Detection Methods

### 3.1. RAA Versus RPA

RAA and RPA share the core principle of recombinase-mediated strand-exchange isothermal nucleic acid amplification. Commercially available RPA kits currently employ the T4 phage-derived UvsX recombinase together with the accessory factors UvsY and the single-stranded DNA binding protein gp32 [40]. According to publicly available information, the formulation descriptions of some commercial RAA kits indicate the use of bacterial/fungal-derived recombinases optimized through protein engineering, whose recombinases may not depend on UvsY-type accessory proteins. However, this description represents manufacturer-disclosed information that lacks independent biochemical validation. For example, some commercially available RAA kits (e.g., from Jiangsu Qitian Gene Technology) list recombinase, SSB, and DNA polymerase as core components, though exact enzyme identities are proprietary. A few diagnostic application studies have reported tolerance of RAA to complex matrices such as feces and blood in specific assay systems [41,42], but these observations were derived from single-assay systems and are insufficient to establish a general tolerance or intrinsic advantage of RAA in complex matrices. An evaluation study of African swine fever virus (ASFV) detection reported that, under specific experimental conditions, the 95% analytical detection limits (LOD_95_) of RAA and RPA, derived from probit analysis, were 53.6 copies/reaction and 93.4 copies/reaction, respectively, with clinical sensitivities of 97.73% and 96.59%, respectively, demonstrating comparable diagnostic performance between the two platforms in that study [43].

### 3.2. RAA Versus Traditional PCR/qPCR

Conventional PCR and real-time quantitative PCR (qPCR) are widely applied nucleic acid detection techniques in pathogen diagnostics and have been established as the benchmark for validating newly developed detection assays in most diagnostic studies. However, these techniques rely on precise thermal cycling procedures and specialized amplification instruments, imposing strict requirements on detection equipment and laboratory conditions. In contrast, RAA operates independently of thermal cycling. It completes nucleic acid amplification within 30 min at a constant temperature of 37–42 °C and greatly reduces equipment dependence, whereas it still exhibits inferior quantitative stability and technical maturity compared with standardized qPCR systems. A real-time fluorescence RAA assay established for the detection of Chinese soft-shelled turtle adenovirus can accomplish detection within 20 min under isothermal conditions, while qPCR maintains more stable performance for the precise quantification of viral loads [44].

### 3.3. RAA Versus LAMP

Loop-mediated isothermal amplification (LAMP) is a classical isothermal amplification technology performed at 60–65 °C, and its amplification results can be determined by turbidity or color changes. However, LAMP requires four to six specific primers targeting multiple conserved regions of the template, which leads to complicated primer design and difficult system optimization [45,46]. In comparison, RAA only requires a single pair of primers to complete amplification at a near-physiological temperature of 37–42 °C, effectively reducing equipment dependence and technical operation thresholds. Notably, RAA does not show comprehensive advantages over LAMP. For example, LAMP may be preferred for applications requiring high tolerance to inhibitors in untreated samples, while RAA may be preferred for applications requiring rapid results at near-ambient temperatures. Open-tube RAA assays present a relatively higher risk of amplicon aerosol contamination and false-positive results, while LAMP exhibits better anti-interference capacity when detecting untreated clinical specimens [47].

### 3.4. RAA Versus ELISA

Enzyme-linked immunosorbent assay (ELISA) is a classical immunological detection technique based on specific antigen–antibody immune reactions. With the advantages of low cost, simple operation and high throughput, ELISA has been widely used for large-scale serological investigations. However, this method fails to detect pathogens at the early infection stage before seroconversion [48] and is difficult to identify latent and low-load infections. In contrast, RAA targets pathogen-specific nucleic acid sequences and enables early diagnosis and trace pathogen detection, presenting improved analytical sensitivity and detection timeliness compared with ELISA serological assays. Nevertheless, RAA requires strict closed-tube operation or closed-cartridge systems to prevent aerosol contamination, and its overall detection throughput is limited compared with the mature ELISA system [3,47].

Table S3 systematically summarizes the core technical characteristics, reaction conditions, and detection performance of five commonly used detection methods in veterinary research, including RAA, RPA, LAMP, qPCR, and ELISA. These techniques differ significantly in biochemical principles, reaction systems, and operational parameters. Overall, each detection method possesses unique technical advantages and inherent limitations; thus, the selection of detection platforms should be flexibly determined according to practical application scenarios, sample types, and specific detection requirements.

## 4. Current Challenges and Future Perspectives

RAA technology presents promising prospects for the rapid detection of veterinary pathogens, while several technical limitations restrict its large-scale clinical application. Veterinary clinical samples, such as feces, whole blood, and tissue homogenates, usually contain complex matrix components. Although RAA tolerates low levels of sample inhibitors, unpurified crude samples may induce matrix interference, leading to fluorescence quenching, decreased polymerase activity, and non-specific amplification, thereby reducing detection accuracy [40]. The development of integrated and user-friendly on-site pretreatment methods, including functionalized magnetic bead purification and simple membrane filtration, is therefore essential to improve the stability and accuracy of field-based RAA detection.

In addition to matrix interference, imperfect quality control systems further hinder the standardized application of veterinary RAA assays. Widespread inhibitory substances in animal samples frequently cause amplification failure and false-negative results, emphasizing the necessity of rigorous internal reference settings and quality control strategies for reliable detection. A standardized RAA detection system requires comprehensive quality control measures, including extraction controls, internal amplification controls, positive controls, no-template controls, and full-process contamination monitoring. Unified criteria for assay validity and standardized anti-contamination protocols are also indispensable. Although dual RAA assays integrated with competitive internal controls have been applied in human pathogen detection [49], systematically validated and mature quality control systems remain lacking for veterinary RAA detection, limiting the reliability and practicability of this technology in animal clinical testing. A minimum quality control panel for veterinary RAA should include: (1) a positive control template at the assay’s LOD, (2) a negative control (no-template control), (3) an internal amplification control (IAC) to monitor inhibition, and (4) an extraction control when nucleic acid extraction is performed. Future methodological optimization of RAA should prioritize the design and validation of complete quality control modules to standardize detection systems and facilitate its clinical promotion.

Furthermore, multiplexing remains limited, typically to only a few targets per single reaction. Spatial isolation strategies, such as microfluidic compartmentalization and droplet digital amplification, can alleviate primer competition and excessive ATP consumption, effectively improving multiplex detection performance [50]. In addition, the development and large-scale preparation of highly stable lyophilized reaction mixes can mitigate enzyme activity instability caused by ambient temperature fluctuations. This optimization improves the whole workflow, covering sample pretreatment, amplification reaction, and result interpretation, enabling RAA to better adapt to epidemiological surveillance in grassroots and resource-limited veterinary settings.

## 5. Conclusions

As a promising molecular diagnostic tool in modern veterinary medicine, RAA is suitable for on-site rapid detection in farms and veterinary clinics. It integrates multiple signal readout formats, including real-time fluorescence, lateral flow dipsticks, and CRISPR-Cas-based signal detection, providing a novel technical option for the surveillance of animal infectious diseases. As an effective complement to conventional detection methods, RAA can cooperate with existing well-established diagnostic systems to improve the overall framework of animal pathogen detection. RAA technology is currently at a technology readiness level (TRL) of 6–7 for many veterinary pathogen detection applications, with validated prototypes demonstrated in relevant field environments. However, widespread adoption requires further standardization and commercialization efforts. Despite its favorable application prospects, further studies involving diversified clinical validation, integrated sample pretreatment establishment, and standardized contamination prevention and quality control procedures are still required to promote the standardized application of RAA in veterinary diagnostics.

## Data Availability

No new data were created or analyzed in this study. Data sharing is not applicable to this article.

## Supplementary Materials

The following supporting information can be downloaded at: https://www.mdpi.com/article/10.3390/microorganisms14102352/s1. Table S1. Performance comparison of various RAA-derived diagnostic platforms for rapid detection of veterinary pathogens; Table S2. Clinical diagnostic performance of published RAA assays for veterinary samples; Table S3. The comparison of different detection methods in the veterinary field.
